# Fabrication of Microstructured Hydrogels via Dehydration for On‐Demand Applications

**DOI:** 10.1002/smll.202406092

**Published:** 2024-10-22

**Authors:** Pang Zhu, Yasindu Wickrama Surendra, Niloofar Nekoonam, Soroush Aziz, Peilong Hou, Sagar Bhagwat, Qingchuan Song, Dorothea Helmer, Bastian E. Rapp

**Affiliations:** ^1^ Laboratory of Process Technology NeptunLab Department of Microsystems Engineering (IMTEK) Albert Ludwig University of Freiburg 79110 Freiburg Germany; ^2^ Freiburg Center of Interactive Materials and Bioinspired Technologies (FIT) Albert Ludwig University of Freiburg 79110 Freiburg Germany; ^3^ Glassomer GmbH 79110 Freiburg Germany; ^4^ Freiburg Materials Research Center (FMF) Albert Ludwig University of Freiburg 79104 Freiburg Germany

**Keywords:** anisotropic wetting surface, dehydration, microlens arrays, microstructured hydrogels, open microfluidics

## Abstract

Microstructured hydrogels show promising applications in various engineering fields from micromolds to anisotropic wetting surfaces and microfluidics. Although methods like molding by, e.g., casting as well as 3D printing are developed to fabricate microstructured hydrogels, developing fabrication methods with high controllability and low‐cost is an on‐going challenge. Here, a method is presented for creating microstructures through the dehydration of double network hydrogels. This method utilizes common acrylate monomers and a mask‐assisted photopolymerization process, requiring no complex equipment or laborious chemical synthesis process. The shape and profile of microstructures can be easily controlled by varying the exposure time and the mask used during photopolymerization. By altering the monomer and the mask used for fabricating the second network hydrogel, both convex and concave microstructures can be produced. To showcase the utility of this method, the patterned hydrogel is utilized as a mold to fabricate a polydimethylsiloxane microlens array via soft lithography for imaging application. In addition, a patterned hydrogel surface exhibiting obvious anisotropic wetting properties and open microfluidic devices which can achieve fast directional superspreading within milliseconds are also fabricated to demonstrate the versatility of the method for different engineering fields.

## Graphical abstract

Hydrogels with microstructured surface are fabricated via dehydration of double network hydrogels utilizing the distinct swelling behaviors between the first network hydrogel and the second network hydrogel in an acid solution. This method can generate uniform convex and concave microstructures with different topographies and profiles, which subsequently can serve as micromolds for soft lithography and open microfluidic platforms.

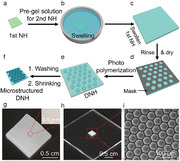

## 1 Introduction

Microstructured hydrogels show promising applications as micromolds,^[^ ^1^ ^]^ for fabricating anisotropic wetting surfaces and microfluidics.^[^ ^2^ ^]^ Various methods have been reported to fabricate hydrogels with microstructured surface. Replication molding with hard molds or soft molds, also called “soft lithography” which uses casting as a straightforward strategy has been widely used to fabricate hydrogels with patterned surface.^[^ ^3^ ^]^ Master molds can be made via lithography or 3D printing,^[^ ^2^, ^3^, ^4^ ^]^ from which a polydimethylsiloxane (PDMS) replica can be cast and then serves as the mold for casting the pre‐gel solution. However, for such casting molding methods, the generation of masters and molds involves cumbersome operation procedures and the demolding process is a persistent problem often causing damages to the hydrogel surface. Strategies like using sacrificial mold materials can facilitate demolding,^[^ ^5^ ^]^ but generation and deconstruction of sacrificial molds consumes significant amounts of resources, as these molds can be only used once and the required dissolving processes are often very slow. Direct methods for generating soft microstructures also exist. As an example, 3D printing based on two‐photon polymerization (TPP) has been established as an emerging technology demonstrating the capacity to fabricate microstructured hydrogel with high precision.^[^ ^6^ ^]^ As an example, Yu et al. printed a polyethylene glycol diacrylate hydrogel with fine microstructures using TPP via green light illumination at low laser thresholds.^[^ ^7^ ^]^ However, due to the linear scanning mode of TPP, this process is time consuming and the high cost of equipment hinders its widespread application.

Another approach for hydrogel structuring is inspired by the self‐growth processes observed in natural organisms, where well‐defined microstructures emerge by absorbing nutrients from the environmental or internal tissues. Researchers have explored methods to grow structures on soft polymer surfaces via external stimuli. As an example, Mu et al. utilized force‐triggered radical polymerization to create microstructures on a double network hydrogel (DNH) surface, employing readily available commercial chemicals.^[^ ^2a^ ^]^ Although their method is straightforward and facile, it results in microstructures with rough and irregular surface topography and relatively lower lateral resolution of about 500 µm. In addition, force as an external stimulus is less controllable and the corresponding indenters have to be fabricated by increasingly intricate processes for small sized indentations. Xue et al. reported a light‐regulated microstructure fabrication method based on dynamic swollen substrates which requires no molds and the heights of microstructures can be conveniently controlled by illumination.^[^ ^8^ ^]^ However, the synthesis of the dynamic substrate is complex and the minimum size of fabricated microstructures is about 350 µm. The high surface roughness of microstructures and irregular profiles are common problems which can be observed in a similar method which achieves simple pattern formation by diffusion of depleted species into illuminated hydrogel areas at a resolution of about 300 µm.^[^ ^9^ ^]^ Next to the self‐growth processes mentioned above, utilizing dehydration to cause changes of internal body microstructures is another common phenomenon in plants to adapt to external environments,^[^ ^10^ ^]^ which also inspires artificial microstructure fabrication. As examples, Oran et al. and Han et al. first activated hydrogel scaffolds via femtosecond laser and deposited different materials like nanoparticles to achieve microstructures insides the hydrogel network after shrinking the hydrogel in an acid solution.^[^ ^11^ ^]^ However, in their work, the hydrogel was only used as a template for assembly of materials to construct micro‐ (or nano‐) structures. To fabricate hydrogels with microstructures on the surface utilizing dehydration has not yet been reported.

In this paper, we introduce a straightforward method for fabricating microstructured hydrogels via dehydration of DNH. This approach requires minimal equipment and relies on readily available common acrylic monomers and initiators. The first network hydrogel (1st NH) primarily comprises of sodium acrylate (SA), which is highly pH‐sensitive and significantly shrinks in a low‐pH solution. The second network hydrogel (2nd NH) is polymerized using mask‐assisted UV illumination, and the 2nd NH exhibits distinct pH responsive behaviors, which shrinks slightly or swells rather than shrinks notably like the 1st NH in a low‐pH environment. The structure topographies and profiles can be controlled by altering the exposure time and the mask pattern during the second photopolymerization. Convex and concave microstructures with a minimum diameter size of 16 and 68 µm, respectively, are fabricated by adjusting the composition of the 2nd NH and photomasks used during the photopolymerization. To showcase the versatility of this method, we fabricated a PDMS microlens array (MLA) via soft lithography, employing the structured DNH as a mold. A microstructured hydrogel surface with anisotropic wetting properties was fabricated which shows distinct contact angles in perpendicular and parallel direction to the microstructures. In addition, microgrooved hydrogels with various patterns of open microchannel were fabricated to achieve fast directional superspreading within hundreds of milliseconds over 5 mm, which show promising applications in microfluidic engineering field.

## 2 Results and Discussion

### 2.1 Fabrication of Convex Microstructured Hydrogel

**Figure 1:**
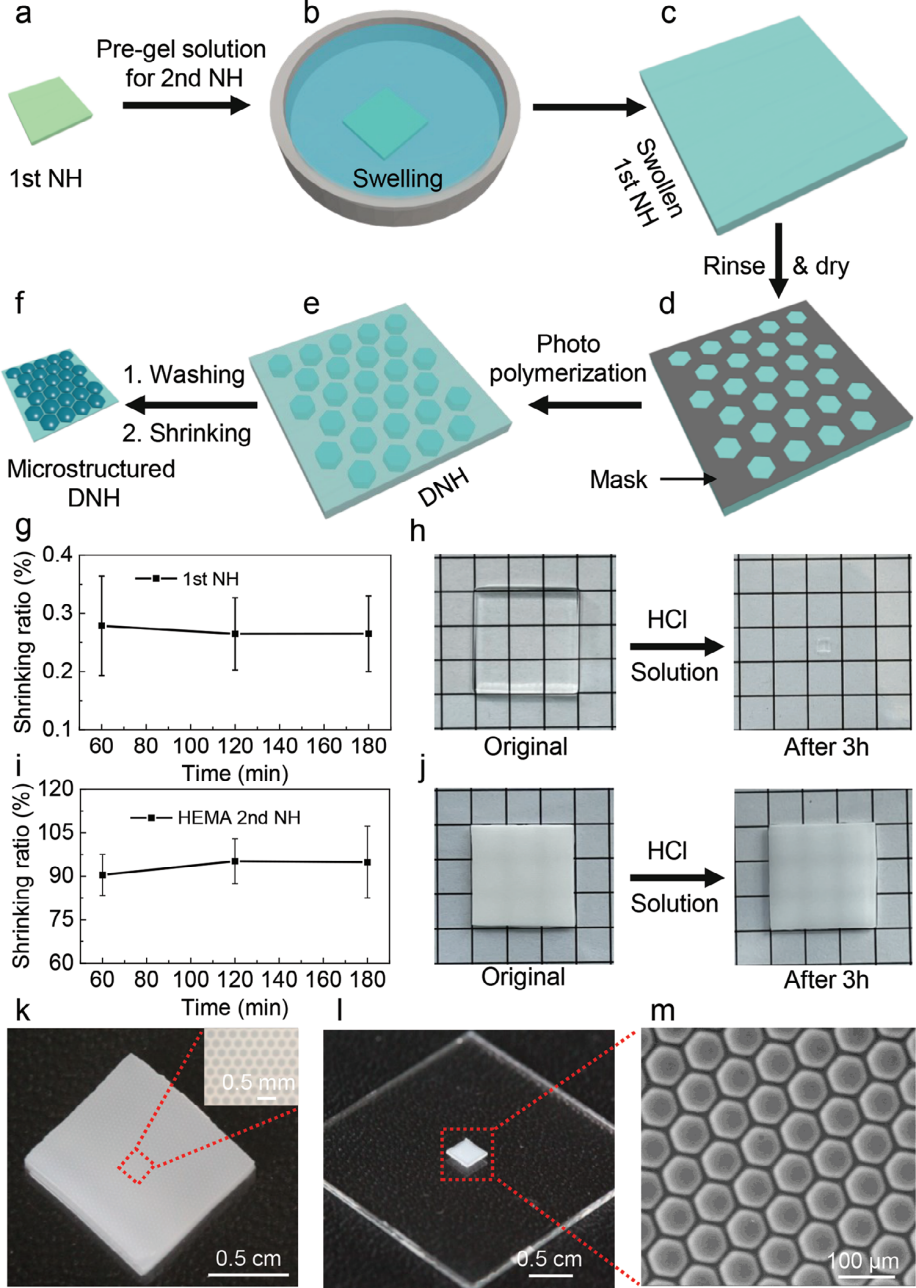
Fabricating microstructured hydrogel via dehydration of the double network hydrogel (DNH). A first network hydrogel (1st NH) based on sodium acrylate (SA) (a) is immersed into a 2‐hydroxyethyl methacrylate (HEMA) pre‐gel solution (b) to reach a fully swollen state (c). d) Photopolymerization of the pre‐gel solution to achieve the second network hydrogel (2nd NH) is conducted using UV illumination under a physical mask, e) which results in a HEMA‐DNH. f) After immersing the DNH into 10 mm HCl solution, the microstructured surface is obtained owing to different shrinkage ratios between the illuminated areas and unilluminated areas. Shrinking behaviors of 1st NH and HEMA 2nd NH in HCl solution were characterized. g) Weight change of the 1st NH: comparison between the hydrogel reaching a fully swollen state in DI water and shrinkage in HCl solution over time. The hydrogel shrinks to around 0.3% of its original weight. h) Pictures of the 1st NH shown in (g) at the fully swollen state in DI water and after immersion in HCl solution for 3 h. i) Weight change HEMA 2nd NH: comparison between the hydrogel reaching a fully swollen state in DI water and shrinkage in HCl solution over time. The hydrogel shrinks to around 95% of its original weight. j) pictures of the HEMA‐SNH HEMA 2nd NH shown in (i) at the fully swollen state in DI water and after immersion in HCl solution for 3 h. k) The optical picture of HEMA‐DNH after photopolymerization via a physical mask with microhexagon patterns (diameter 170 µm, gap 100 µm, Figure S17a, Supporting Information) and the microscope image of the structure (inset). l) Shrunken HEMA‐DNH with microstructured surface and m) environmental scanning electron microscope (E‐SEM) image of the DNH shown in (h). Data in (g) and (i) are presented as mean values ± standard deviation (SD). Error bars represent the SD from three samples.

The detailed strategy to fabricate microstructures on hydrogel surface based on the dehydration of DNH is illustrated in **Figure** **1**. First, the 1st NH (Figure 1a, 0.3 mm thickness) which primarily consists of sodium acrylate was prepared via thermal radical polymerization. Concerning the thickness of the 1st NH, spacers with three thickness (0.1, 0.3, and 1.0 mm) were used to prepare the 1st NH. However, 0.1 mm samples were too thin after shrinking and could not be handled. For the thicker samples of 1.0 mm samples, after swelling in the pre‐gel solution, the thickness increased dramatically to about 4.5 mm, which caused easy breaking of the gel during processing and thus could also not be handled. Therefore, it was concluded that 0.3 mm samples were most suitable for the project. The 1st NH was cut into pieces of ≈4 × 4 mm^2^ and immersed into the pre‐gel solution of the 2nd NH (Figure 1b) which consists mainly of 2‐hydroxyethyl methacrylate (HEMA). The 1st NH then absorbs the pre‐gel solution. The maximum swollen size is reached after ≈1 h (Figure 1c and Figure S1, Supporting Information), but the samples were regularly kept in the pre‐gel solution for 24 h before using. The swollen 1st NH samples were taken out and rinsed with deionized (DI) water and dried using a nitrogen (N_2_) gun to eliminate any residual liquid on the surface. To define microstructures, the hydrogel was illuminated by UV illumination through a microstructured mask (Figure 1d) creating the HEMA‐DNH (Figure 1e). The HEMA‐DNH was rinsed thoroughly with DI water and subsequently immersed into DI water for 24 h in order to remove unpolymerized materials. Lastly, to achieve desired microstructures, the HEMA‐DNH was immersed into 10 mm hydrochloric acid (HCl) solution. Since the 1st NH is highly sensitive to pH, it undergoes significant shrinkage (shrinking to about 0.3% of its initial weight, see Figure 1g,h) in the HCl solution.^[^ ^11a^ ^]^ The 2nd NH composed of HEMA is not pH‐sensitive and experiences minimal change with a weight shrinking ratio of about 95% in the HCl solution (Figure 1I,j). In addition, the 2nd HEMA NH is very soft and shows quite low storage modulus of only about 200 Pa at 0.1 rad s^−1^ (Figure S2, Supporting Information). As a result, the HEMA‐DNH shrinks and the photopolymerized hydrogels is squeezed out forming final microstructures (Figure 1f and Figure S3, Supporting Information). To further illustrate the process, Figure 1k shows the HEMA‐DNH after photopolymerization using a physical mask with a microhexagon array (diameter 170 µm, gap 100 µm, Figure S17a, Supporting Information). The initially transparent 1st NH transformed into a whitish appearance due to the formation of the 2nd NH composed of HEMA. The polymerized structure is already observed on the DNH in this state, as shown in the inset of Figure 1k. After being immersed into the HCl solution, the DNH shrinks obviously and the final microstructured HEMA‐DNH was obtained (Figure 1l). The surface topography of the shrunken hydrogel was characterized using an environmental scanning electron microscope (E‐SEM) (Figure 1m).

### 2.2 Optimization and Versatility of Microstructured Hydrogel Fabrication Method

**Figure 2:**
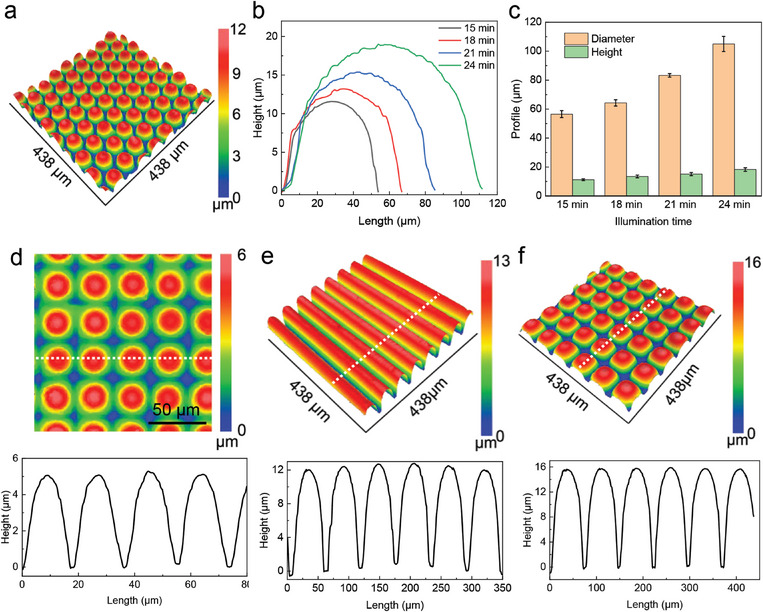
Convex microstructures on the HEMA‐DNH. a) 3D topography of the microhexagon array (mask size: diameter 170 µm, gap 100 µm, Figure S17a, Supporting Information) shown in Figure 1m obtained via white light interferometry (WLI). b,c) Profile and corresponding quantification of WLI data of microhexagon arrays (mask size: diameter 170 µm, gap 100 µm, Figure S17a, Supporting Information) produced with different exposure times in the photopolymerization step. d) Profile of a microcircle array (mask size: diameter 20 µm, gap 80 µm, Figure S17b, Supporting Information) of microstructured HEMA‐DNH with a minimum diameter of 16.6 ± 0.8 µm; further microstructures on HEMA‐DNH showing e) a microstrip array (mask size: width: 100 µm, gap 100 µm, Figure S17c, Supporting Information) and f) a microsquare array (mask size: length 150 µm, gap 100 µm, Figure S17d, Supporting Information). Data in (c) is presented as mean values ± standard deviation (SD). Error bars represent the SD from three samples.

The microstructure profile shown in Figure 1l was further characterized using WLI (**Figure** **2**a). The fabricated microhexagon structures exhibited uniformity with a hexagon diameter of 56.5 ± 2.4 µm and height of 11.5 ± 0.6 µm (Figure S4, Supporting Information). To explore the impact of varying exposure times on the resulting microstructures, the profiles of microstructures fabricated under different exposure times were analyzed. The exposure times were chosen between 15 and 24 min, as shorter exposure was not sufficient for the formation of microstructures and longer illumination led to rough surface structures (see Figure S5, Supporting Information). As exhibited in Figure 2b,c, extended exposure times led to an increase in both the diameter of the microhexagon structure (from 56.5 ± 2.4 to 105 ± 5.2 µm) and the height (from 11.2 ± 0.6 to 18.2 ± 1.2 µm). This phenomenon is attributed to the greater degree of polymerization within the 2nd NH formed during longer illumination, making it more resistant to compression during the subsequent shrinking process. To prove this, we investigated the rigidity of the HEMA single network hydrogel (HEMA‐SNH) via rheology tests, as shown in Figure S6, Supporting Information, the storage modulus increased from about 200 to 700 Pa at 0.1 rad s^−1^ by increasing illumination time from 15 to 24 min. In addition, the weight of the dried HEMA‐DNH after different illumination times was investigated (Figure S7, Supporting Information). At longer exposure times, the hydrogel retained a greater mass, which also indicates a higher degree of polymerization of the 2nd NH. Furthermore, over polymerization is a common issue in photolithography, to clarify its effect on the size of final microstructures, for this effect on the initial hexagonal sizes (demonstrated in Figure 1e,k) of the HEMA‐DNH illuminated with 24 min was checked immediately after photo polymerization using microscope (Figure S8, Supporting Information), and it demonstrates that the microhexagon size remains constant with the micropatterns of the employed mask (diameter 170 µm, gap 100 µm, Figure S17a, Supporting Information), indicating no overcuring during the second photo polymerization process.

To test the limit of resolution of the presented method, we employed a physical mask with a microcircle array (diameter 20 µm, gap 80 µm, Figure S17b, Supporting Information), to fabricate small structures with a diameter of 16.6 ± 0.8 µm and a height of 4.8 ± 0.5 µm (Figure 2d).This is beyond the resolution of microstructures fabricated on soft substrates reported recently,^[^ ^2^, ^8^ ^]^ and microstructured hydrogels printed with common 3D printing technology, like digital light processing.^[^ ^12^ ^]^ To verify the versatility of our strategy, microstructures with diverse configurations were fabricated by employing photomasks with distinct patterns including microstrip patterns and microsquare patterns (Masks: see Figure S17c,d, Supporting Information). As illustrated in Figure 2e,f, HEMA‐DNHs structured with a microstripe array and a microsquare array were prepared and demonstrated uniform profiles.

### 2.3 Fabrication of Concave Microstructured Hydrogel

**Figure 3:**
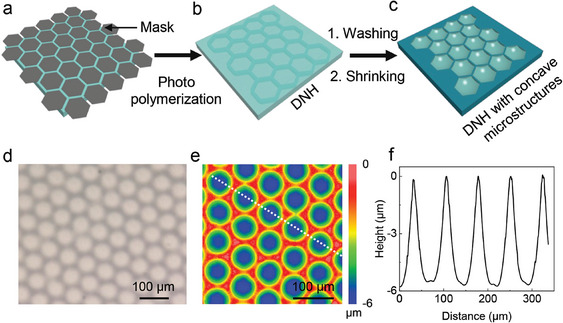
Fabrication of concave microstructures through dehydration of NIPAM‐DNH. Schematic illustration of fabricating concave microstructures: a) The 1st NH is covered with a negative mask with microhexagon patterns (diameter 50 µm, gap 20 µm, Figure S17e, Supporting Information). b) After illumination, a polymerized NIPAM‐DNH is obtained. c) The final NIPAM‐DNH with concave microstructures is achieved by immersing the NIPAM‐DNH into 10 mm HCl solution. d) A microscopy image of the polymerized NIPAM‐DNH. e) A WLI image of the 2D topography of the concave microstructures and f) corresponding cross sectional profiles showing concave structures of 68.8 ± 2.7 µm diameter and 5.5 ± 0.5 µm depth.

Except for convex microstructures, hydrogels with concave microstructures also exhibit a great importance in engineering fields.^[^ ^13^ ^]^ Here, as shown in **Figure** **3**, by adjusting the pre‐gel solution and photomask employed to construct the 2nd NH, concave microstructures can be fabricated using the described method. Similar to fabricating convex microstructures shown in Figure 1a–c, the 1st NH hydrogel was cut into small pieces (about 4 × 4 mm^2^) and immersed into a pre‐gel solution based on N‐isopropylacrylamide (NIPAM). Here, NIPAM is chosen as the monomer instead of HEMA to construct the 2nd NH and it is because that poly(*N*‐isopropylacrylamide) (PNIPAM, the polymer derived from NIPAM) swells slightly in low‐pH solutions (swollen weight ratio: 105%, see Figure S9, Supporting Information) and shows higher rigidity (about 2600 Pa at 0.1 rad s^−1^, see Figure S10, Supporting Information) compared with HEMA‐SNH, which is the key for fabricating concave microstructures. In this way, after being immersed into the acid solution, the 2nd NH works as a frame (Figure 3b) to constrain the shrinkage of the 1st NH in *X*–*Y* direction, but in *z*‐direction there is a notable shrinkage in the nonilluminated area resulting in concave microstructures (Figure 3c and 3D profile: see Figure S11, Supporting Information). We employed a photomask with large black microhexagon patterns (50 µm diameter) and narrow transparent gaps (20 µm width) (Figure S17e, Supporting Information) to fabricate the concave microstructures. As shown in Figure 3d, the microscopy image of the NIPAM‐DNH reveals the successful polymerization and embedding of the 2nd NIPAM NH (dark hexagons) within the 1st NH. Following dehydration of the NIPAM‐DNH in the 10 mm HCl solution, we obtained well‐defined microhexagon concave microstructures with a diameter of 68.8 ± 2.7 µm and a depth of 5.5 ± 0.5 µm (Figure 3e,f).

### 2.4 Optical Application to Fabricate a Microlens Array

**Figure 4:**
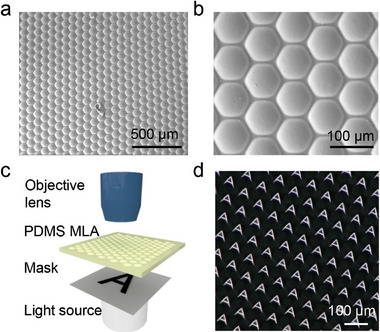
A microlens array (MLA) fabricated by replication of PDMS from the microstructured HEMA‐DNH. a,b) SEM images of the PDMS MLA under different magnifications. c) Schematic illustration of the setup for imaging performance test. d) Uniform images obtained from the replicated PDMS MLA showing the versatility of the microstructure fabrication method for optical applications.

To exemplify practical applications of our strategy, we employed the convex microstructured HEMA‐DNH as a mold for fabricating a PDMS MLA via soft lithography as shown in **Figure** **4**. The microstructured HEMA‐DNH shown in Figure 1f,m was used for replication. To fabricate the PDMS MLA, the HEMA‐DNH was dried using a N_2_ gun to remove remaining water on the hydrogel surface, liquid PDMS was poured onto the surfaces and cured for 24 h under room temperature. The PDMS mold was passivated with a silane solution and the final positive PDMS MLA was obtained by curing liquid PDMS in the passivated mold. The PDMS MLA shows high surface quality according to SEM characterization (Figure 4a,b), and the surface smoothness was determined via atomic force microscopy (AFM) with an *R*_a_ value of 27.2 ± 6.4 nm (Figure S12, Supporting Information). The setup used for assessing the imaging performance was equipped with an objective lens and a charge‐coupled device camera (Figure 4c). A black film showing a transparent letter “A” was placed between the PMDS MLA and a light source. Figure 4d shows that each microlens of the PDMS MLA can project a sharp image (Video S1, Supporting Information). This demonstrates the high surface quality and uniformity of the PDMS MLA, and the potential application of the microstructure fabrication method for optical engineering.

### 2.5 Anisotropic Wetting Surface and Open Microfluidic Platform

**Figure 5:**
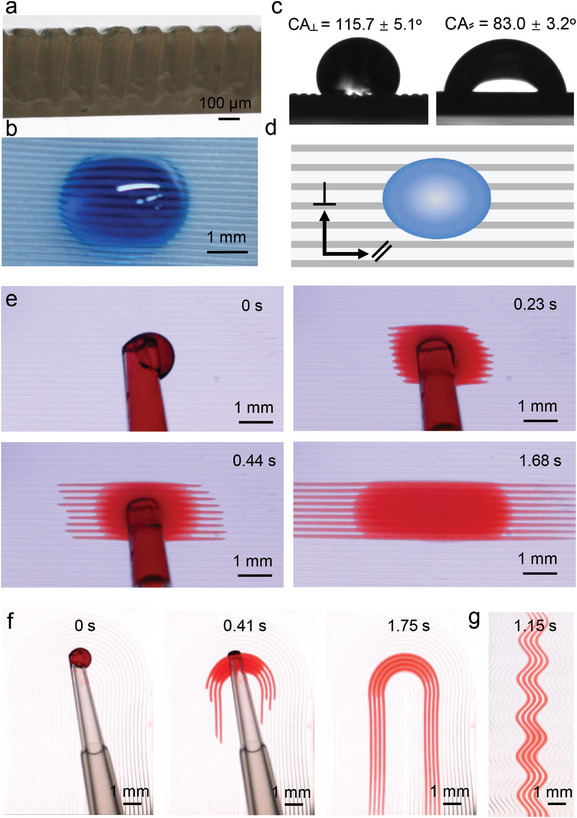
Anisotropic wetting surface and open microfluidic platforms for directional superspreading. a) Cross‐sectional topography of NIPAM‐DNH with grooved microstructure (groove size: width 63 ± 9 µm, depth 39 ± 7 µm). b) A dyed water droplet (5 µL) on the NIPAM‐DNH surface shown in Figure 5a demonstrates anisotropic wetting behaviors. c,d) Contact angles at directions which are perpendicular and parallel to the microgrooves and corresponding schematic illustration. e) The directional superspreading process of a water droplet on microstructured AM‐DNH with straight microchannels (channel size: width 67 ± 8 µm, depth 36 ± 6 µm). f) U‐shaped (channel size: width 98 ± 11 µm, depth 49 ± 9 µm) and g) S‐shaped microchannels (channel size: width 133 ± 17 µm, depth 62 ± 13 µm). Data in (c) is presented as mean values ± standard deviation (SD). Error bars represent the SD from three samples.

Another application of our method is to fabricate surface with anisotropic wetting properties. As shown in **Figure** **5**a, a microstructured NIPAM‐DNH was fabricated using a physical mask with a microstrip array (width 100 µm, gap 100 µm, Figure S17c, Supporting Information). The width and depth of the microgrooves on the final hydrogel surface were 63 ± 9 and 39 ± 7 µm. Owing to the less hydrophilicity of PNIPAM resulting from methyl groups of NIPAM monomer and surface microstructures, the droplet rested on the microstructured surface with an elliptical shape. For the static contact angle test (Figure 5f,g), the microstructured NIPAM‐DNH exhibits obvious anisotropic wetting properties with a contact angel (CA_⊥_) of 115.7° ± 5.1° in the direction perpendicular to the microgrooves and a contact angle (CA_≈_) of 83.0° ± 3.2° in the direction parallel to the microgrooves. While the flat NIPAM‐DNH shows lower water contact angle values (67.5° ± 6.2°) and isotropic wetting behaviors (Figure S13a,b, Supporting Information). Another common application for such stripe microstructures is for directional superspreading, which is particularly important for point‐of‐care diagnostics and disposable biosensors.^[^ ^2c^ ^]^ To show our method's versatility to fabricate such open microfluidics, we engineered a material system to ensure the formation of hydrophilic open microchannels with our technology. To achieve this, an acrylamide (AM)‐based pre‐gel solution is used to construct the 2nd NH and it is because that polyacrylamide (PAM, the polymer derived from AM) shows high rigidity (storage modulus: 11 kPa at 0.1 rad s^−1^, see Figure S14, Supporting Information) as well as high hydrophilicity with a water contact angle of 25.8° ± 4.3° allowing the water droplet to spread on the hydrogel surface (see Figure S15, Supporting Information) which is critical for directional superspreading. AM‐DNH with microgroove structures were fabricated using a mask with strip array patterns (width 100 µm, length 100 µm, Figure S17c), the width and depth of the open microchannels are about 67 ± 8 and 36 ± 6 µm (Figure S16, Supporting Information). As soon as the water droplet contacted the microgrooved hydrogel surface, it spread along the microchannels immediately and the flow was constrained in the initial spreading channels without overflowing into other microchannels, exhibiting excellent performance of directional superspreading behavior (Figure 5e and Video S2, Supporting Information). To further illustrate the possibility of our strategy to develop open microfluidic platforms, we designed microchannels with different morphologies, like S‐shaped and U‐shapes (masks: see Figure S17g,h, Supporting Information). When the water droplet was placed onto the hydrogel surfaces, both hydrogels exhibited excellent directional superspreading phenomenon by guiding water flow along the curved microchannels (Figure 5f,g and Videos S3 and S4, Supporting Information).

## 3 Conclusion

In summary, we introduced a straightforward method for the fabrication of surface microstructured hydrogels through the dehydration of DNH. This method offers exceptional accessibility, eliminating the need for complex equipment and intricate chemical synthesis. The process relies on swelling a 1st NH in a pre‐gel solution of a 2nd NH and consecutively polymerizing the pre‐gel solution to achieve a DNH. The DNH is washed and then treated with a HCl solution to achieve the final microstructure. The morphology and profile of the fabricated microstructures can be easily controlled by adjusting the exposure time and masks employed during photopolymerization. We showed the production of both convex and concave microstructures by adjusting the monomer composition and photomasks used for fabricating the 2nd NH. Using the structured HEMA‐DNH as a mold, we fabricated a PDMS MLA via soft lithography showing exceptional surface quality and imaging performance. By employing monomer NIPAM to construct the 2nd NH, surfaces with anisotropic wetting properties can be achieved. In addition, we fabricated microfluidic devices with various patterns of microchannels based on microgrooved AM‐DNH for fast directional superspreading. Owing to the straightforward mechanism, simplified procedures and high controllability, we believe that this method will promote applications of hydrogels in various engineering fields.

## 4 Experimental Section

### Materials

AM (99%), HEMA (99%), 2‐hydroxy‐4′‐(2‐hydroxyethoxy)−2‐methylpropiophenone (Irgacure 2959, 98%), *N*,*N*’‐methylenebis(acrylamide) (MBA, 99%), and sodium acrylate (SA) were purchased from Sigma‐Aldrich, Germany. Ammonium peroxydisulphate (APS, 98%), isopropanol (99.5%), and toluene (99.5%) were purchased from Carl Roth, Germany. 1*H*,1*H*,2*H*,2*H*‐perfluoroctyldimethylchlorosilane (97%) was purchased from Abcr GmbH, Germany. NIPAM (98%) was purchased from Tokyo Chemical Industry (TCI), Deutschland GmbHGermany. PDMS (601A and 601B) was purchased from Wacker, Germany. Transparent silicon rubber sheet (0.1 and 0.3 mm) was purchased from Technikplaza, Germany.

### Preparation of the First Network Hydrogel (1st NH)

The 1st NH was fabricated via thermal radical polymerization. Typically, SA (5.161 mmol), AM (1.615 mmol), MBA (0.034 mmol), and APS (0.061 mmol) were added into DI water (4 mL) and the mixture was put into the ultrasonic bath for 5 min under room temperature to get a transparent pre‐gel solution. The obtained solution was then injected into a glass cell composed of two hydrophobic glass slides and a spacer made from a silicon rubber sheet (0.1 and 0.3 mm thickness) and cured in an oven under 45 °C for 24 h to achieve a 1st NH.

### Preparation of Pre‐Gel Solutions

Three pre‐gel solutions were developed for the immersion of the 1st NH. To fabricate concave microstructures, a HEMA‐based pre‐gel solution was prepared consisting of HEMA (32.98 mmol), MBA (1.027 mmol), Irgacure 2959 (0.892 mmol), and DI water (20 mL). To fabricate concave microstructures and anisotropic wetting surfaces, a NIPAM‐based pre‐gel solution was prepared consisting of NIPAM (26.511 mmol), MBA (1.297 mmol), Irgacure 2959 (0.892 mmol), and H_2_O (20 mL). To fabricate open microfluidic platforms, an AM‐based pre‐gel solution was prepared consisting of AM (42.206 mmol), MBA (0.649 mmol), Irgacure 2959 (0.892 mmol), and H_2_O (20 mL).

### Preparation of the DNH with Microstructures

The 1st NH was cut into small pieces (about 4 × 4 mm^2^) and immersed into one of the pre‐gel solutions and kept in the dark for 24 h before usage. To polymerize the 2nd NH, the fully swollen 1st NH was quickly rinsed with 15 mL DI water and dried using a N_2_ gun to remove remaining liquid from the hydrogel surface. A regular glass cover slide (thickness: 100 µm) was placed onto the 1st NH surface and a physical mask was placed onto the glass cover slide. The prepared system was placed under a UV lamp (320–400 nm) to polymerize the 2nd NH. Physical masks and exposure time were changed to achieve microstructures with different shapes and profiles. After UV illumination, the polymerized DNH was rinsed with DI water and immersed into DI water for 24 h. The water was exchanged five to six times to wash out the unpolymerized materials. Depending on the pre‐gel solution used for the 2nd network, the obtained DNH was named as HEMA‐DNH, NIPAM‐DNH, or AM‐DNH according to which monomer was used to construct the DNH. The DNH were then immersed into 10 mm HCl solution overnight to obtain the final microstructures.

### Fabrication of HEMA‐, NIPAM‐, and AM‐SNH

For analysis of the properties of the hydrogel materials, single network hydrogels were prepared from the HEMA‐, NIPAM‐, and AM‐based pre‐gel solutions. The HEMA‐based pre‐gel solution was injected into a glass cell composed of two hydrophobic glass slides and a spacer made from the silicon rubber sheet (0.3 mm thick) and then illuminated under UV lamp for 15 min to achieve a polymerized hydrogel. The resulting hydrogel is denoted as HEMA‐SNH. The NIPAM‐based pre‐gel solution was injected into a glass cell composed of two hydrophobic glass slides and a spacer made from the silicon rubber sheet (0.3 mm thick), and then illuminated under a UV lamp for 30 min to achieve a polymerized hydrogel. The resulting hydrogel is denoted as NIPAM‐SNH. The AM‐SNH was prepared from the AM‐based pre‐gel solution in the same way as the NIPAM‐SNH.

### Preparation of Hydrophobic Glass Slides

To prepare the glass cell for gel polymerization, the glass slide surface was first modified. For this, glass slides were immersed into HCl‐Methanol solution (volume ratio: 50%−50%) for 30 min, followed by flushing using DI water and isopropanol, glass slides were dried using a N_2_ gun and then immersed into the 1*H*,1*H*,2*H*,2*H*‐perfluoroctyldimethylchlorosilane solution (2.9 vol% in toluene) for 1 h, after being flushed using DI water and isopropanol and dried with a N_2_ gun, hydrophobic glass slides were obtained.

### Determination of Weight Changes of Hydrogels Immersed into Solutions

Swelling and shrinking behaviors of the hydrogels in solution were determined by weight analysis. The wet hydrogel was weighed (*W*_0_) and then the hydrogel was immersed into the corresponding solutions. The weight of hydrogel was measured at different timepoints after immersion (*W*_t_). The weight ratio was calculated according to the following equation: weight ratio = *W*_t_/*W*_0_×100%.

In order to investigate the influence of illumination time on the polymerization degree, the remaining weight ratio of dried HEMA‐DNHs polymerized at different illumination times was tested. The weight of HEMA‐DNH which was washed in water for 24 h after polymerization was measured and defined as *W*_0_. Then the HEMA‐DNH was immersed into 10 mm HCl solution overnight and dried completely in air. Afterward, the weight was measured and defined as *W*_dry_. The remaining weight ratio was calculated according to the following function: weight ratio = *W*_dry_/*W*_0_ ×100%.

### Rheological Measurement

To investigate the influence of illumination on the rigidity of the hydrogel, rheological assessment was carried out. Frequency sweep tests (0.1–100 rad s^−1^) were performed on a stress‐controlled rheometer MCR310 (Anton Paar, Germany). Samples were tested at 20 °C using a parallel plate geometry (diameter: 25 mm) at a fixed strain of 1% which is in the linear viscoelastic regime of samples as determined by amplitude sweep measurements.

### Fabrication of PDMS Microlens Array

Gels for replication were stored in the shrinking solution and only taken out immediately prior to the replication process. To fabricate PDMS MLA, the microstructured HEMA‐DNH was first placed on a glass slide and dried using a N_2_ gun to remove remaining liquid from the hydrogel surface. The liquid PDMS was mixed and degassed according to the manufacturers recommendation and poured onto HEMA‐DNH surface and left to cure for 24 h at room temperature. The cured PDMS was peeled off from the HEMA‐DNH and treated with air plasma (Atto Plasma cleaner, Diener electronic, Germany) for 5 min to activate the surface, and immersed into 1*H*,1*H*,2*H*,2*H*‐perfluoroctyldimethylchlorosilane solution (2.9 vol% in toluene) for 10 min. The functionalized PDMS was washed using isopropanol and dried in the oven under 100 °C for 30 min. Then the prepared PDMS liquid was poured onto the surface‐modified PDMS mold and cured for 24 h to get the final PMDS MLA.

### Characterization

The hydrogel surface was characterized using an E‐SEM (Quanta 250, FEI, USA) and a WLI (Newview 9000, Zygo, Germany). The replicated PDMS surface topography was characterized via a scanning electron microscope (SEM, Tescan Amber X, Czech Republic). The surface smoothness was determined via an AFM (JPK Nanowizard 4, Bruker, Germany). Static water contact angles were measured under the room temperature (about 20 °C) using an OCA 15EC contact angle goniometer (DataPhysics, Germany) and calculated with SCA20 software by carefully placing a water droplet (2 µL) onto the hydrogel surface.

### Statistics Analysis

Three samples were measured to characterize microstructures’ topography and size, hydrogels’ swelling/shrinking behaviors and modulus, and water contact angle of the hydrogel surfaces. The results are presented with the mean values and standard deviations. Data were analyzed by Microsoft Excel and Origin program.

## Conflict of Interest

The authors declare no conflict of interest.

## Supporting information



Supporting Information

Supplemental Video 1

Supplemental Video 2

Supplemental Video 3

Supplemental Video 4

## Data Availability

The data that support the findings of this study are available from the corresponding author upon reasonable request.

## References

[1] a) T.‐D. Dang , Y. H. Kim , J. H. Choi , G.‐M. Kim , J. Micromech. Microeng. 2012, 22, 015017.

[2] a) Q. Mu , K. Cui , Z. J. Wang , T. Matsuda , W. Cui , H. Kato , S. Namiki , T. Yamazaki , M. Frauenlob , T. Nonoyama , M. Tsuda , S. Tanaka , T. Nakajima , J. P. Gong , Nat. Commun. 2022, 13, 6213. 36266283 10.1038/s41467-022-34044-8 PMC9585076

[3] a) T. Yu , C. K. Ober , Biomacromolecules 2003, 4, 1126. 12959574 10.1021/bm034079m

[4] a) C. He , X. Chen , Y. Sun , M. Xie , K. Yu , J. He , J. Lu , Q. Gao , J. Nie , Y. Wang , Y. He , Bio‐Des. Manuf. 2022, 5, 641.

[5] a) A. P. Golden , J. Tien , Lab Chip 2007, 7, 720. 17538713 10.1039/b618409j

[6] a) J. Xing , J. Liu , T. Zhang , L. Zhang , M. Zheng , X. Duan , J. Mater. Chem. B 2014, 2, 4318. 32261570 10.1039/c4tb00414k

[7] H. Yu , H. Ding , Q. Zhang , Z. Gu , M. Gu , Adv. Manuf. 2021, 24, 4266.

[8] L. L. Xue , X. H. Xiong , B. P. Krishnan , F. Puza , S. Wang , Y. J. Zheng , J. X. Cui , Nat. Commun. 2020, 11, 963. 32075979 10.1038/s41467-020-14807-x PMC7031321

[9] J. E. Stumpel , D. Q. Liu , D. J. Broer , A. Schenning , Chemistry 2013, 19, 10922. 23821576 10.1002/chem.201300852

[10] a) T. Hagihara , M. Toyota , Plants 2020, 9, 577. 32375332 10.3390/plants9050587 PMC7284940

[11] a) D. Oran , S. G. Rodriques , R. Gao , S. Asano , M. A. Skylar‐Scott , F. Chen , P. W. Tillberg , A. H. Marblestone , E. S. Boyden , Science 2018, 362, 1281. 30545883 10.1126/science.aau5119 PMC6423357

[12] M. Caprioli , I. Roppolo , A. Chiappone , L. Larush , C. F. Pirri , S. Magdassi , Nat. Commun. 2021, 12, 2462. 33911075 10.1038/s41467-021-22802-z PMC8080574

[13] a) K. C. Hribar , D. Finlay , X. Ma , X. Qu , M. G. Ondeck , P. H. Chung , F. Zanella , A. J. Engler , F. Sheikh , K. Vuori , S. C. Chen , Lab Chip 2015, 15, 2412. 25900329 10.1039/c5lc00159e PMC4439309

