# Supplementary material for: Fabrication of Microstructured Hydrogels via Dehydration for On‐Demand Applications
Source: Small. 2024 Oct 22;20(52):2406092. doi: 10.1002/smll.202406092 (PMC11673557; doi:10.1002/smll.202406092)
Supplement: Supplementary file 1 — Supporting Information [file SMLL-20-2406092-s002.docx]

Supporting Information

Fabrication of Microstructured Hydrogels via Dehydration for on-demand Applications

Pang Zhu, Yasindu Wickrama Surendra, Niloofar Nekoonam, Soroush Aziz, Peilong Hou, Sagar Bhagwat, Qingchuan Song, Dorothea Helmer*, Bastian E. Rapp


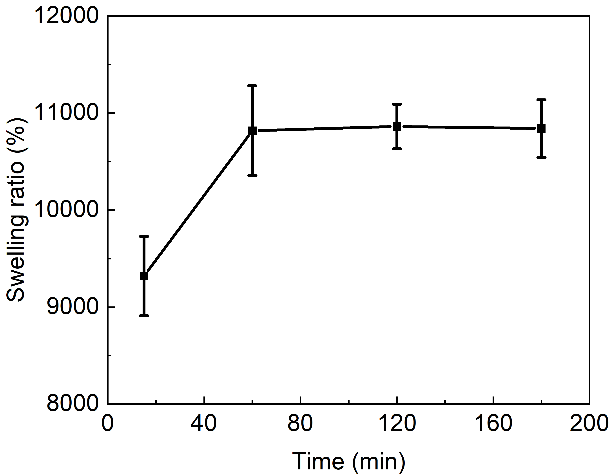


**Figure S1.** Swelling behavior of the 1st NH in the HEMA based pre-gel solution was investigated via weight change: comparison between the 1st NH freshly prepared and swelling in HEMA based pre-gel solution over time. An increase of approximately 11000% by weight was detected over the first 60 min. Data is presented as mean values ± standard deviation (SD). Error bars represent the SD from three samples.


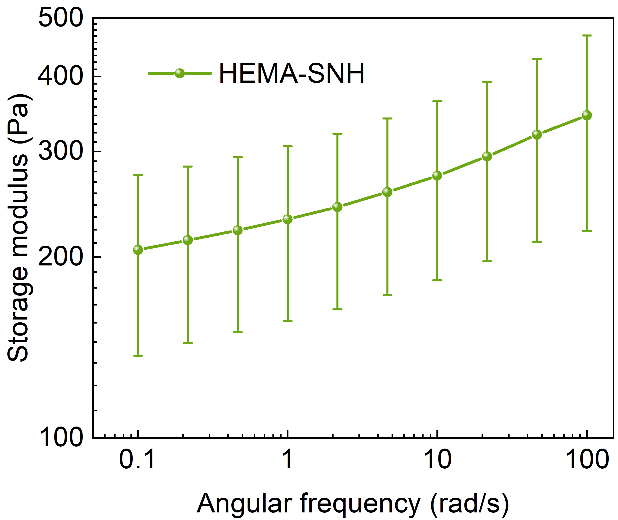


**Figure S2.** Dynamic rheology of HEMA-SNH prepared with 15 min illumination showing elastic modulus G′ as functions of the angular frequency with a G′ of about 200 Pa at 0.1 rad/s. Data is presented as mean values ± standard deviation (SD). Error bars represent the SD from three samples.


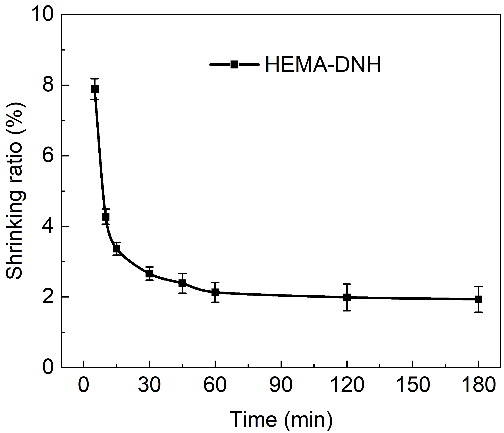


**Figure S3.** Shrinking behavior of HEMA-DNH shown in Figure 1g in HCl solution was investigated via weight change: comparison of the HEMA-DNH reaching a fully swollen state in deionized water and shrinkage in HCl solution over time. The hydrogel reaches an equilibrium after approximately 2 h and shrinks to around 2.0% of its original weight. Data in is presented as mean values ± standard deviation (SD). Error bars represent the SD from three samples.


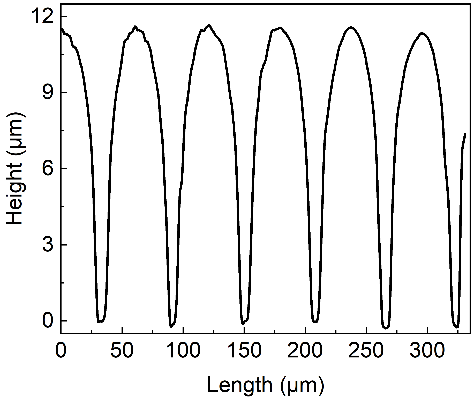


**Figure S4.** The cross-sectional profile of the microstructured HEMA-DNH (mask size: diameter 170 µm, gap 100 µm, Figure S14 a) shown in Figure 2a.


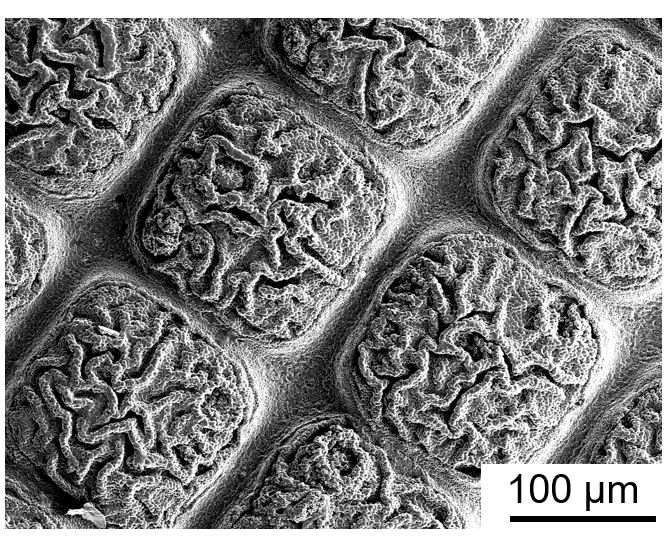


**Figure S5.** The SEM image of microstructured HEMA-DNH polymerized at an exposure time of 40 min under a physical mask with a microsquare array (mask size: length 200 µm, gap 100 µm, Figure 14f). Such long exposure times led to rough microstructures.


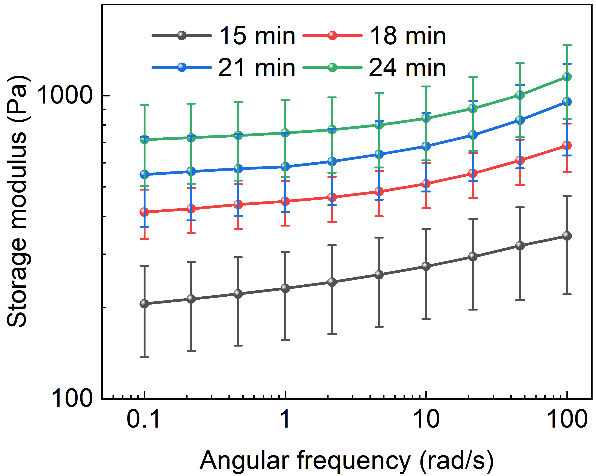


**Figure S6.** Dynamic rheology of HEMA-SNHs prepared with different illumination time showing storage modulus G′ as functions of the angular frequency. The storage modulus of the gels increases upon longer illumination time, indicating a higher degree of polymerization. Data is presented as mean values ± standard deviation (SD). Error bars represent the SD from three samples.


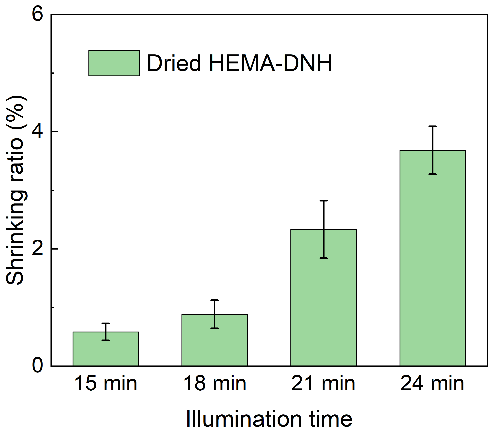


**Figure S7.** Shrinking behavior of HEMA-DNHs polymerized at different exposure time shown in Figure 2b was investigated via weight change: comparison between the wet HEMA-DNH reaching a fully swollen state in DI water and completely dried state. The data shows that at higher exposure times, more monomer polymerizes into the polymer network thus increasing the weight of the dried HEMA-DNH. Data is presented as mean values ± standard deviation (SD). Error bars represent the SD from three samples.


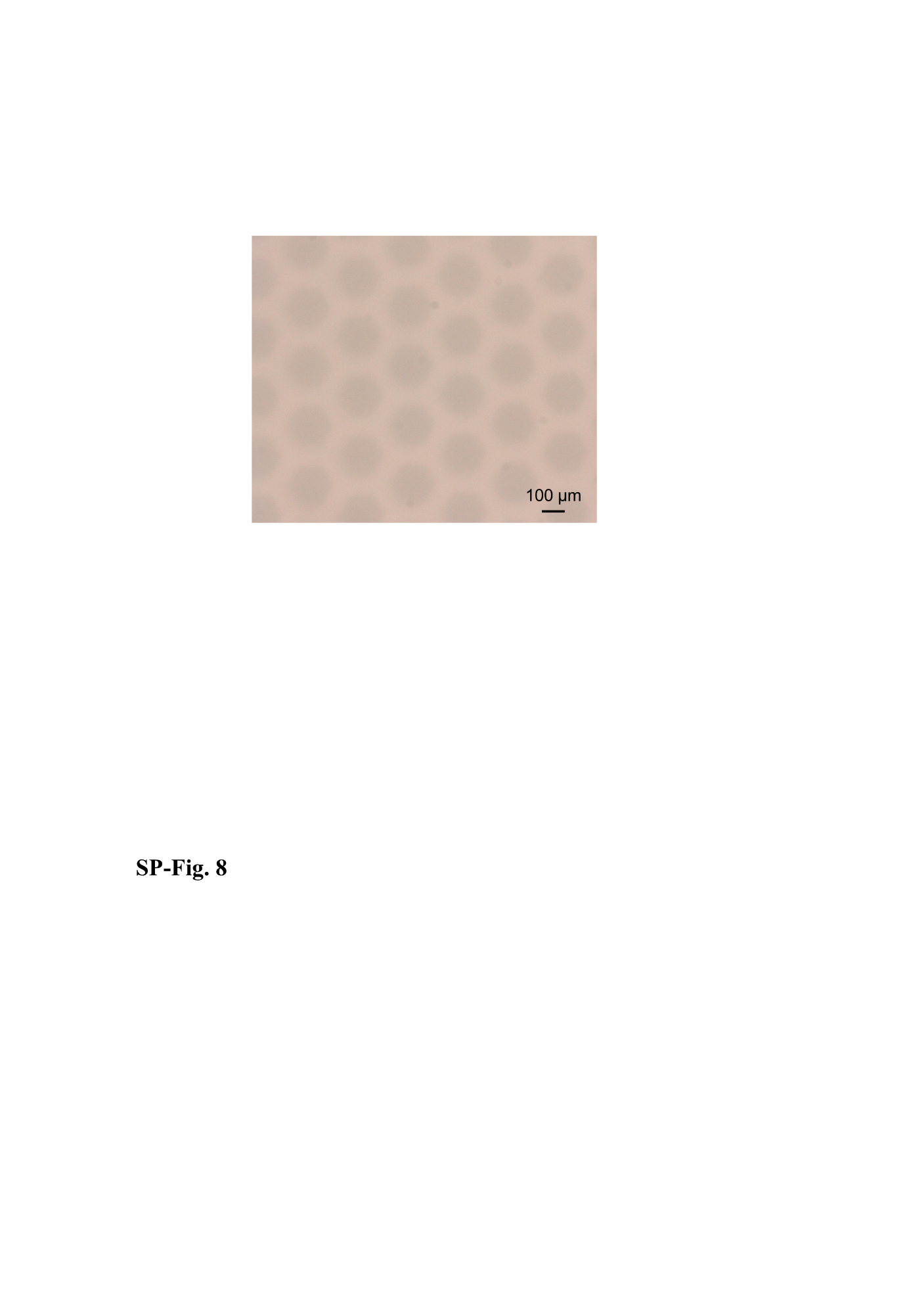


**Figure S8.** The microscope image of HEMA-DNH illuminated with 24 min. The initial microhexagon size is constant with micropatterns of employed photo mask (diameter 170 µm, gap 20 µm, Figure S17 a) implying no over curing during the 2nd photo polymerization.


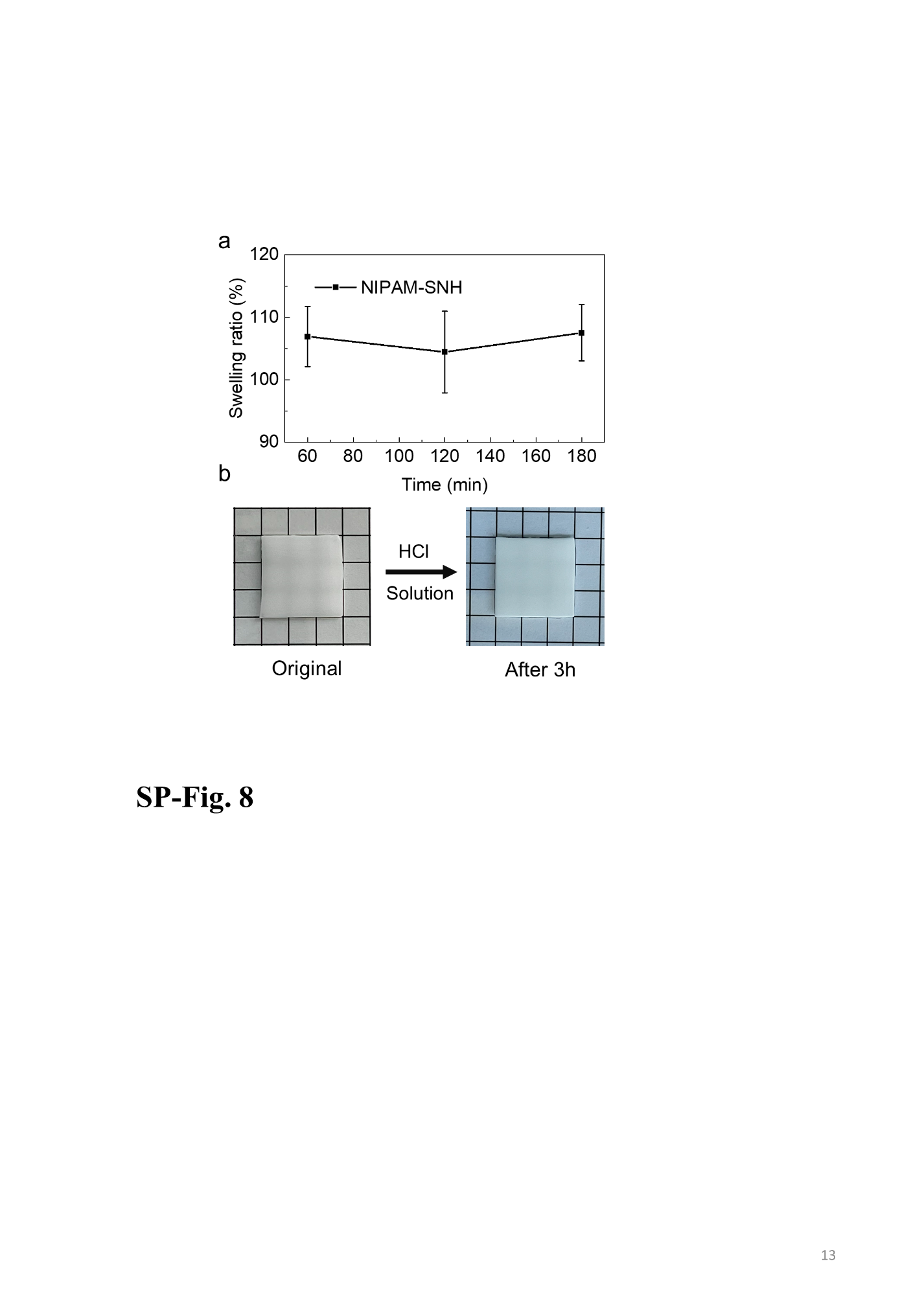


**Figure S9.** Swelling behavior of NIPAM-SNH in HCl solution. (a) Weight change of the NIPAM-SNH: comparison between the hydrogel reaching a fully swollen state in DI water and shrinkage in HCl solution over time. The hydrogel swells to around 105% of its original weight; (b) pictures of the NIPAM-SNH at the fully swollen state in DI water and after immersion in HCl solution for 3 h corresponding to the data shown in (a). Data is presented as mean values ± standard deviation (SD). Error bars represent the SD from three samples.


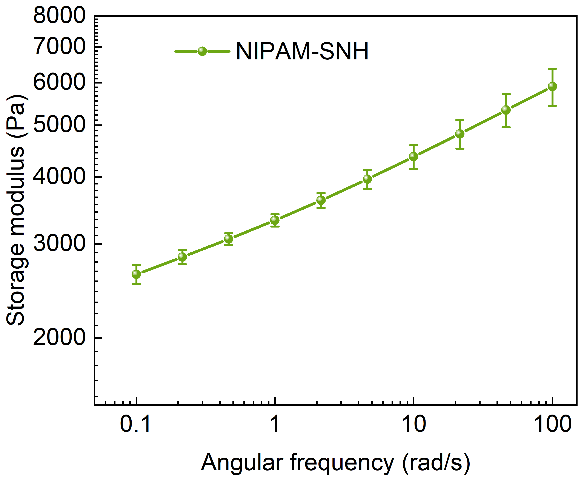


**Figure S10.** Dynamic rheology of NIPAM-SNH prepared storage modulus G′ as functions of the angular frequency with a G′ of about 2600 Pa at 0.1 rad/s. Data is presented as mean values ± standard deviation (SD). Error bars represent the SD from three samples.


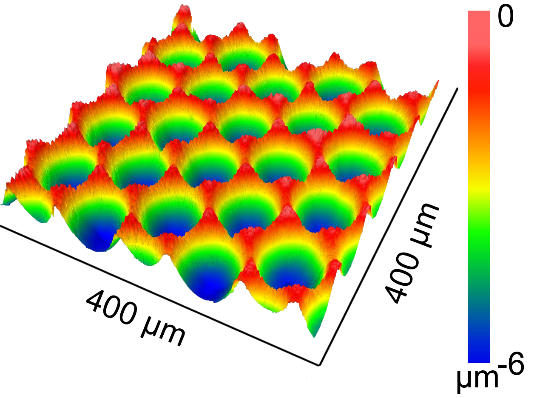


**Figure S11** 3D topography of concave microstructures (mask size: diameter 50 µm, gap 20 µm, Figure S17 e) shown in Figure 3e obtained via white light interferometry (WLI).


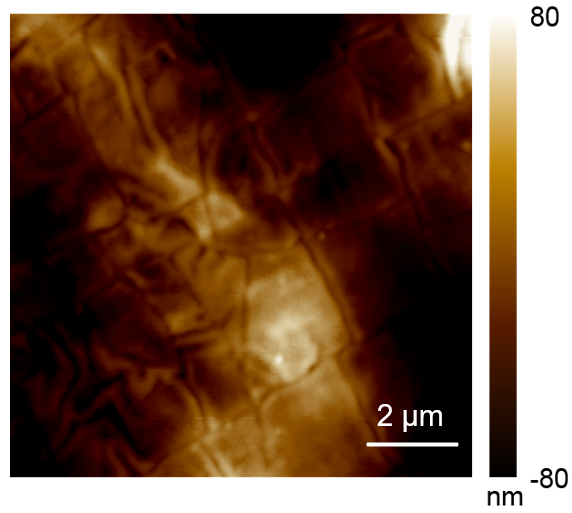


**Figure S12.** Surface roughness analysis of the PDMS MLA (shown in Figure 5) fabricated using microstructured HEMA-DNH polymerized at an exposure time of 15 min (shown in Figure 1) as a mold via soft lithography. The image shows an AFM scan of lateral size 10 µm.


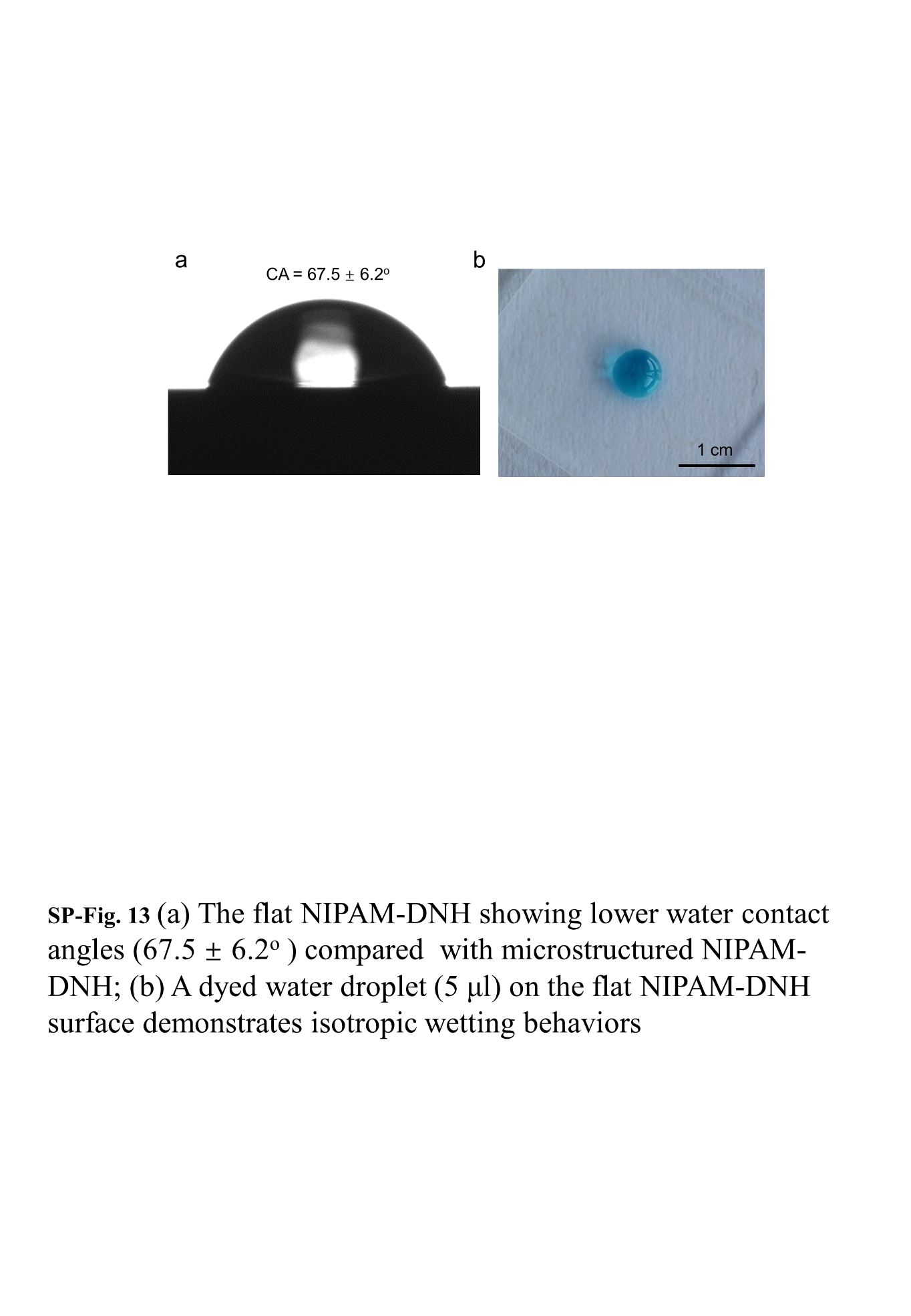


**Figure S13.** (a) The flat NIPAM-DNH showing lower water contact angles (67.5 ± 6.2^o^) compared with microstructured NIPAM-DNH; (b) A dyed water droplet (5 μl) on the flat NIPAM-DNH surface demonstrates isotropic wetting behaviors.


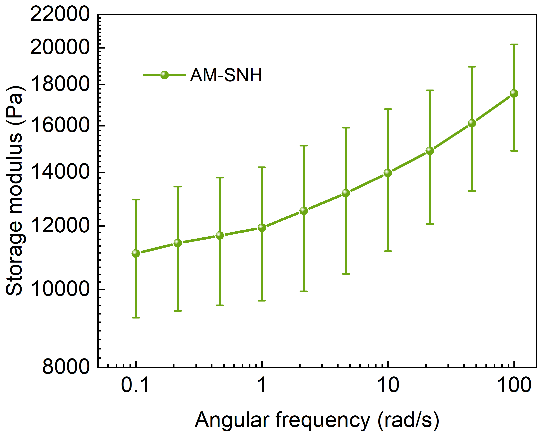


**Figure S14.** Dynamic rheology of AM-SNH showing storage modulus G′ as functions of the angular frequency with a G′ of about 11000 Pa at 0.1 rad/s. Data is presented as mean values ± standard deviation (SD). Error bars represent the SD from three samples.


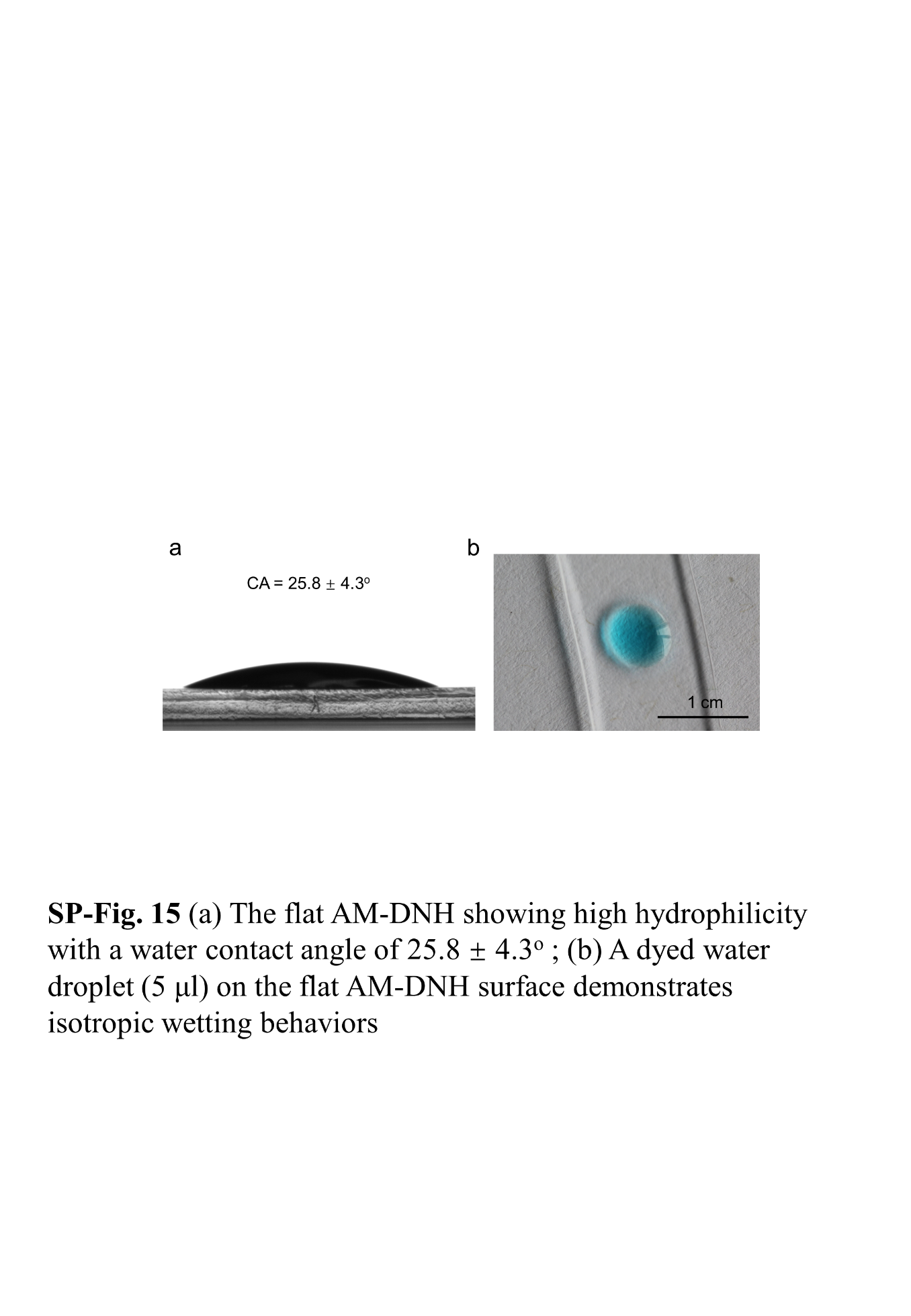


**Figure S15**. (a) The flat AM-DNH showing high hydrophilicity with a water contact angle of 25.8 ± 4.3^o^ ; (b) A dyed water droplet (5 μl) on the flat AM-DNH surface demonstrates isotropic wetting behaviors.


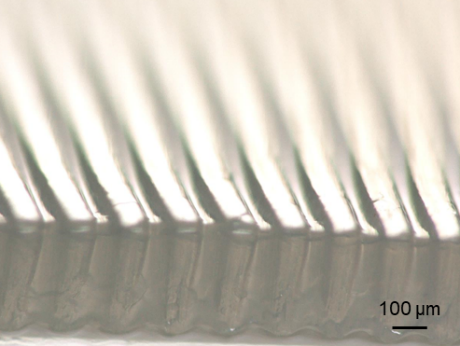


**Figure S16.** The cross-sectional topography of the open microfluidic made from AM-DNH (shown in Figure 5e) with straight microchannels for directional superspreading.


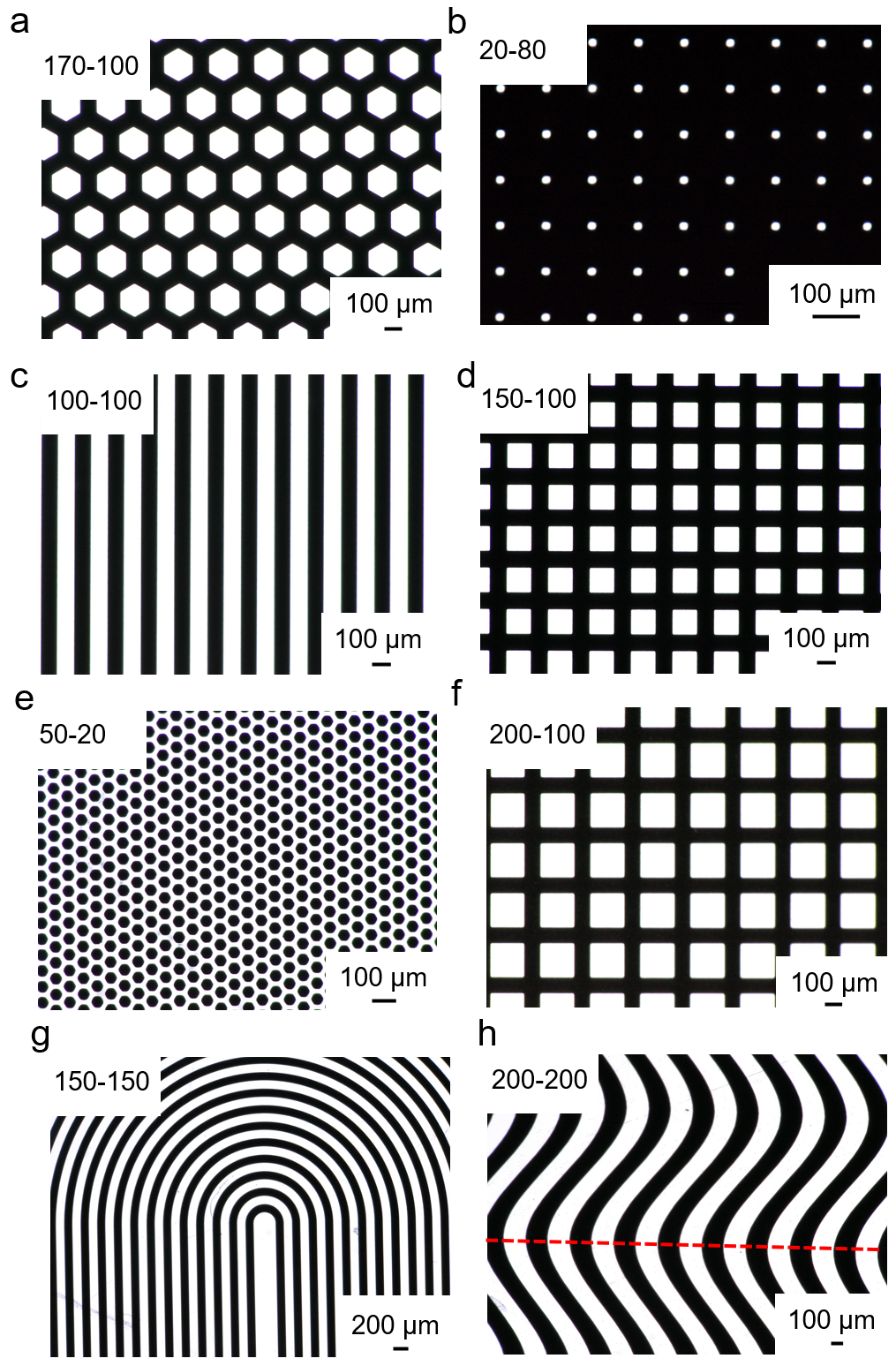


**Figure S17.** Physicals masks employed to fabricated microstructures. (a) A microhexagon array: diameter: 170 μm, gap 100 μm; (b) A microsquare array: diameter 20 μm, gap 80 μm; (c) A microstrip array: width 100 μm, gap 100 μm ; (d) A microsquare array: length 150 μm, gap 100 μm; (e) A negative microhexagon array: diameter: 50 μm, gap 20 μm; (f) A microsquare array: length 200 μm, gap 100 μm; (g) A U-shape pattern: width 150 μm, gap 150 μm; (h) A S-shape pattern: width 200 μm, gap 200 μm along the marked red dash line.
